# The Alteration of Intrinsic Excitability and Synaptic Transmission in Lumbar Spinal Motor Neurons and Interneurons of Severe Spinal Muscular Atrophy Mice

**DOI:** 10.3389/fncel.2019.00015

**Published:** 2019-02-07

**Authors:** Jianli Sun, Melissa A. Harrington

**Affiliations:** ^1^Delaware Center for Neuroscience Research, Delaware State University, Dover, DE, United States; ^2^Department of Biological Science, Delaware State University, Dover, DE, United States

**Keywords:** spinal muscular atrophy, intrinsic excitability, synaptic transmission, motor neuron, interneuron

## Abstract

Spinal muscular atrophy (SMA) is the leading genetic cause of death in infants. Studies with mouse models have demonstrated increased excitability and loss of afferent proprioceptive synapses on motor neurons (MNs). To further understand functional changes in the motor neural network occurring in SMA, we studied the intrinsic excitability and synaptic transmission of both MNs and interneurons (INs) from ventral horn in the lumbar spinal cord in the survival motor neuron (SMN)Δ7 mouse model. We found significant differences in the membrane properties of MNs in SMA mice compared to littermate controls, including hyperpolarized resting membrane potential, increased input resistance and decreased membrane capacitance. Action potential (AP) properties in MNs from SMA mice were also different from controls, including decreased rheobase current, increased amplitude and an increased afterdepolarization (ADP) potential. The relationship between AP firing frequency and injected current was reduced in MNs, as was the threshold current, while the percentage of MNs showing long-lasting potentiation (LLP) in the intrinsic excitability was higher in SMA mice. INs showed a high rate of spontaneous firing, and those from SMA mice fired at higher frequency. INs from SMA mice showed little difference in their input-output relationship, threshold current, and plasticity in intrinsic excitability. The changes observed in both passive membrane and AP properties suggest greater overall excitability in both MNs and INs in SMA mice, with MNs showing more differences. There were also changes of synaptic currents in SMA mice. The average charge transfer per post-synaptic current of spontaneous excitatory and inhibitory synaptic currents (sEPSCs/sIPSCs) were lower in SMA MNs, while in INs sIPSC frequency was higher. Strikingly in light of the known loss of excitatory synapses on MNs, there was no difference in sEPSC frequency in MNs from SMA mice compared to controls. For miniature synaptic currents, mEPSC frequency was higher in SMA MNs, while for SMA INs, both mEPSC and mIPSC frequencies were higher. In SMA-affected mice we observed alterations of intrinsic and synaptic properties in both MNs and INs in the spinal motor network that may contribute to the pathophysiology, or alternatively, may be a compensatory response to preserve network function.

## Introduction

The molecular genetics underlying spinal muscular atrophy (SMA) have been well established since 1995 with the discovery that it results from deletion or mutation of the *survival motor neuron 1* (*SMN1*) gene encoding the survival motor neuron (SMN) protein (Lefebvre et al., 1995). The human genome contains two SMN-coding genes, *SMN1* and *SMN2* (Lefebvre et al., 1995; Rochette et al., 2001). The telomeric *SMN1* expresses full-length SMN protein while the centromeric *SMN2* predominantly produces the unstable SMNΔ7 protein due to the aberrant splicing of exon 7 (Lorson et al., 1999; Monani et al., 2000). Deletion of, or mutations in *SMN1* are found in all types of SMA patients, with the severity of disease depending on the copy number of *SMN2*, which contributes different levels of residual SMN protein (Gennarelli et al., 1995; Lefebvre et al., 1995).

The SMN protein is ubiquitously expressed and localized in nuclear “gems” (Gubitz et al., 2004), in the cytoplasm (Liu and Dreyfuss, 1996), in neuronal growth cones (Fan and Simard, 2002), and neuronal axon (Fallini et al., 2010). The SMN protein plays an essential role in the assembly of the spliceosomal small nuclear ribonucleoproteins (Gubitz et al., 2004), regulates RNA metabolism (Li et al., 2014), actin cytoskeleton dynamics (Hensel and Claus, 2018), mRNA transport (Donlin-Asp et al., 2016), ubiquitin homeostasis (Groen and Gillingwater, 2015), bioenergetics pathways (Boyd et al., 2017) and synaptic vesicle release (Kong et al., 2009). Unfortunately, it is still not clear how low levels of SMN protein lead to the pathophysiology associated with SMA.

The most advanced clinical trials for SMA are focused on increasing SMN protein level by upregulating the production of full length protein from the *SMN2* gene (d’Ydewalle and Sumner, 2015), and the first, very costly, drug to specifically treat SMA was approved by the U.S. Food and Drug Administration in December 2016 (Aartsma-Rus, 2017), 125 years after the first description of this disease. However, we still lack a comprehensive therapy for SMA, since increasing SMN level does not abrogate but simply slows down the neurodegenerative process. Additional compounds or approaches that could slow the decline in neuromuscular function would be a major advance for SMA patients and their families. While current treatments and exploratory approaches seek to increase survival based on increasing SMN expression, the development of complementary approaches to preserving neuromuscular function will require a deeper understanding of the molecular pathology underlying the disease process.

Although planning and initiation of movement take place in the cortex, the basal ganglia, midbrain, and hindbrain, the generation of locomotion in vertebrates is mainly determined by neural networks in the spinal cord. Spinal circuits contain the basic instructions for coordinating the sequence of muscle activation during locomotion and are engaged by descending and ascending supraspinal systems for volitional tasks (Arber, 2012; Miri et al., 2013). With the combination of electrophysiology and mouse genetics to identify and manipulate the activity of components of the spinal locomotor networks, in the past two decades, great advances have been made in understanding of the organization of spinal networks in mammals, particularly those for locomotion (Kiehn, 2016).

This network appears to be active at birth, long before locomotion begins (Whelan et al., 2000; Juvin et al., 2007), and during a period when mouse models for SMA show minimal signs of pathology. Recent studies showed the impairment of proprioceptive synaptic input to motor neurons (MNs) observed in SMA mouse models occurs before neuronal loss (Mentis et al., 2011), and loss of vesicular glutamate transporter (VGLUT)1-labeled inputs to MNs has even been detected in embryos (Tarabal et al., 2014). In contrast, an increase of VGLUT1 and Vesicular acetylcholine transporter (VAChT) expression was found in calbindin-immunoreactive interneurons (INs)—Renshaw cells in a mouse model of SMA (Thirumalai et al., 2013). These studies suggest that spinal circuit dysfunction may be a feature of SMA, even though the locomotor activity appears to be normal in P4–6 SMA mice (Thirumalai et al., 2013), and the contribution of spinal motor network to the pathophysiology of SMA cannot be excluded without careful studies. In addition, there is substantial overlap and intermingling between spinal INs for different muscles and motor activities (Barthélemy et al., 2006; Levine et al., 2012).

SMA-related dysfunctions in sensory-motor pathways have been observed to occur prior to neuromuscular junction deficits or cell death, suggesting that there may be an early disease phase in which network function is altered while neurons remain viable. While alterations in MNs’ intrinsic properties have been reported, whether there are changes in the intrinsic and synaptic properties of the various INs involved in the motor network is unknown.

In the current study, we investigated the passive membrane properties, action potentials (APs), long-lasting plasticity in intrinsic excitability, spontaneous and miniature synaptic currents in ventral horn alpha MNs and INs in spinal cord slices from SMA and control mice. We found increased excitability and synaptic abnormality in both types of neurons. The differences in the long-lasting plasticity of the intrinsic excitability and spontaneous synaptic release in both neuron types suggests that there is a compensatory adjustment occurring in SMA. Whether this modulation is beneficial or harmful to the motor output and MN survival is yet to be determined.

## Materials and Methods

### Animals

This study was carried out in accordance with the recommendations of the Institutional Animal Care and Use Committee of Delaware State University. The protocol was approved by the Institutional Animal Care and Use Committee of Delaware State University. Mice were maintained under a 14/10 h light/dark photoperiod with PMI rodent diet (Animal Specialties and Provisions) and water available *ad libitum*. *Smn1* heterozygotes male and female mice of the FVB.Cg-Grm7<Tg(SMN2)89Ahmb >Smn1<tm1Msd > Tg(SMN2*delta7)4299 strain obtained from Jackson Laboratory (stock#: 005025, Bar Harbor, ME, USA) were mated to produce pups for experiments. Postnatal pups from 6 to 10 days old were used in the experiment. Genotyping of mouse pups and adults was done by Transnetyx (Germantown, TN, USA). Pups were genotyped and assigned to experimental groups after recording and data analysis were completed. Wild type and heterozygous pups were pooled as control, pups with the homozygous mutation formed the SMA group as described previously (Zhang et al., 2010).

### Chemicals and Solutions

Artificial cerebral spinal fluid (ACSF) contained (in mM): 126 NaCl, 3 KCl, 2 MgCl_2_, 2 CaCl_2_, 1.25 NaH_2_PO_4_, 10 glucose, and 26 NaHCO_3_ (pH 7.2, 300–310 mOsm). Tissue slicing solution contained (in mM): 2.5 KCl, 10 MgSO_4_, 0.5 CaCl_2_, 1.25 NaH_2_PO_4_, 234 sucrose, 11 glucose, and 26 NaHCO_3_ (pH 7.2, 310–330 mOsm). Intracellular solution contained (in mM): 130 K-gluconate, 10 Hepes, 11 EGTA, 2.0 MgCl_2_, 2.0 CaCl_2_, 4 Na-ATP, and 0.2 Na-GTP (pH 7.2–7.3 with KOH, 290–310 mOsm). Receptor antagonists (in μM) 10 CNQX (6-cyano-7-nitroquinoxaline), 50 LD-AP-5 [D-(−)-2-amino-5-phosphonovaleric acid], 10 bicuculline, and 10 strychnine were included in the ACSF to block AMPA, NMDA, GABA_A_, and glycine receptors respectively. Tetrodotoxin (TTX; 1 μM) was applied to block AP for recording of miniature synaptic currents. All drugs were purchased from Tocris (Bristol, UK), except strychnine which was purchased from Sigma-Aldrich (St. Louis, MO, USA).

### Spinal Cord Slice Preparation

A single mouse of postnatal day P6–P10 was anesthetized in a sealed chamber containing the inhalation anesthetic Isoflurane in an upward flowing Biological Safety Level (BSL) 2 cabinet. After decapitation, the skin and limbs were quickly removed leaving part of the hind limbs for insect pins, and the tissue was immersed in ice-cold oxygenated slicing solution. Two cuts through the shoulders and down the chest cage removed most tissue leaving just the vertebral column and enough ribs on each side for insect pins to hold. The cervical and top thoracic section of the cord were cut off to isolate the intact lumbar segment of the spinal cord. Remaining viscera and tissue around the vertebral column were removed on both dorsal and ventral sides to make it easier for laminectomy. The vertebral column was transferred to a silicon-filled petri dish, pinned dorsal side up with insect pins, and continuously perfused with carbogen-bubbled (5% CO_2_, 95% O_2_) ice-cold slicing solution. A laminectomy on the dorsal side exposed the spinal cord, using fine forceps the dura was peeled along the rostro-caudal axis, and the lumbar enlargement was isolated. Using a transfer pipet, the spinal cord was placed in a groove cut in a block of 5% agar to make a “hot dog”. The surface was dried with filter paper, the agar block was glued onto the cutting chamber block, and 400 μM slices were cut with a LEICA VT1200S tissue slicer (Leica Microsystems, Wetzlar, Germany). Slices were moved into the incubation chamber with a transfer pipet and kept at 34°C for 30 min. Slices were left at room temperature for more than 30 min before recording.

### Whole-Cell Patch-Clamp Recording

The recording chamber was continuously perfused with pre-heated 34°C ACSF at a rate of 2–3 ml/min. MNs and INs were identified using an IR-1000 infrared sensitive camera (DAGE MTI, Michigan City, IN, USA) coupled with a DIC-equipped BX51WI microscope from Olympus (Center Valley, PA, USA). Alpha MNs, which are selectively lost after SMA (Powis and Gillingwater, 2016), were recognized by their minimum soma diameter (>20 μM) and location (lamina IX). INs were distinguished by their smaller minimum soma diameter (<10 μM), high input resistance (>100 MΩ), and depolarized resting membrane potentials (≈ −50 mV). Most INs recorded were located in laminae VII and VIII. Cells were patched in the whole-cell configuration with patch pipettes (resistance of 2–4 MΩ) filled with intracellular solution. Current-clamp recordings were acquired with a MultiClamp 700B amplifier, low pass filtered at 10 kHz and digitized at 100 kHz with a Digidata 1440A (both from Molecular Devices; Sunnyvale, CA, USA). Spontaneous membrane potentials were recorded for 2 min to measure the resting membrane potential (Vrest) and firing frequency for neurons with spontaneous firing. To study AP properties, APs were evoked every 5 s with positive current injection step intervals of 20 pA and 200 pA for 5 ms for INs and MNs, respectively. To study the firing frequency vs. injected current (F-I) relationships, 500 ms negative and positive steps were applied every 10 s with the same step intervals as for AP activation. The membrane potential of the INs was held at −55 mV, and MNs at −65 mV by manually adjusted DC current injection.

Studies of models for neuronal plasticity suggested that memory traces can be supported not only by selective changes in the synaptic strength–synaptic plasticity, but that modifications in neuronal excitability—a plasticity in intrinsic properties, might also contribute to cellular substrates for memory. Long-lasting plasticity in intrinsic properties shares common features with synaptic plasticity which has been more extensively studied (Daoudal and Debanne, 2003). To study long-lasting plasticity in the intrinsic electrical properties of the spinal cord neurons, INs were manually held at −50 mV, and MNs at −60 mV. The test protocol comprised 11 steps total, five negative and five positive, 500 ms duration at 10 s intervals, with the most positive step evoking 12–15 spikes. The conditioning protocol involved a 50 ms positive current injection to evoke three APs in each neuron. For the baseline before conditioning, three test trials were recorded with a 20 s delay, and a total recording of 6 min. For the conditioning stimulation, the conditioning protocol was delivered for 6 min at 2 Hz. Fifteen test trials totaling 30 min of time were recorded immediately after conditioning. For the baseline condition, 20 test trials totaling 40 min were recorded without conditioning stimulation.

Synaptic currents were recorded under voltage-clamp mode. Signals were digitized at 100 KHz, and low pass filtered at 4K and 6K Hz for INs and MNs, respectively. All neurons were clamped at −55 mV to record both excitatory and inhibitory postsynaptic currents (EPSCs and IPSCs) which were recorded from the same neuron simultaneously for 3 min. The outward IPSCs and inward EPSCs currents were confirmed by glutamatergic, GABAergic and glycinergic receptor antagonists (Figure 8A). The liquid junction potential (−13.5 mV) was compensated, and access resistance was continuously monitored and rechecked after each recording. If the series resistance increased by 20% at any time, the recording was terminated or data excluded from analysis.

**Figure 1:**
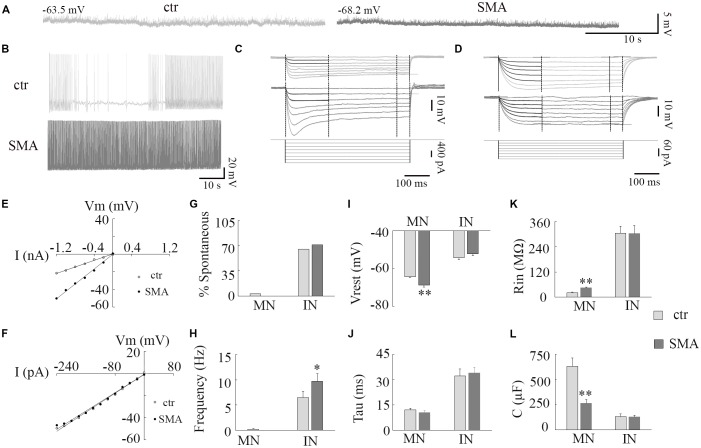
Resting membrane properties of motor neurons (MNs) and interneurons (INs) in survival motor neuron (SMN)Δ7 mouse model mice. **(A)** Representative traces of spontaneous membrane potential in control (left) and spinal muscular atrophy (SMA; right) MNs. **(B)** Representative spontaneous firing in INs of control (top) and SMA (bottom). **(C,D)** Representative traces of membrane potentials evoked by hyperpolarization current pulses (bottom) for calculations of input resistance (Rin), time constant (Tau), and membrane capacitance (C) for control (top) and SMA (middle) MNs **(C)** and INs **(D)**. **(E,F)** Voltage/current relationship for MNs **(E)** and INs **(F)**. **(G)** Percent of neurons spontaneously firing at rest in MNs (*n* = 31 cells for control and 19 cells for SMA) and INs (*n* = 43 cells for control and *n* = 23 cells for SMA). **(H)** Frequency of spontaneous firing at rest for a single MN (from control) and INs (*n* = 28 cells for control and *n* = 23 cells for SMA). **(I)** Resting membrane potential for MNs (*n* = 31 cells for control and *n* = 19 cells for SMA) and INs (*n* = 43 cells for control and *n* = 31 cells for SMA). **(J–L)** Time constant (Tau, **J**), Input resistance (Rin, **K**), Membrane capacitance (C, **L**) in MNs and INs from control (*n* = 19 cells) and SMNΔ7 (*n* = 13 cells). *P < 0.05, **P < 0.01 with student *t*-test. Overall cells were recorded from spinal cords of eight SMA mice and 11 of their littermate controls.

**Figure 2:**
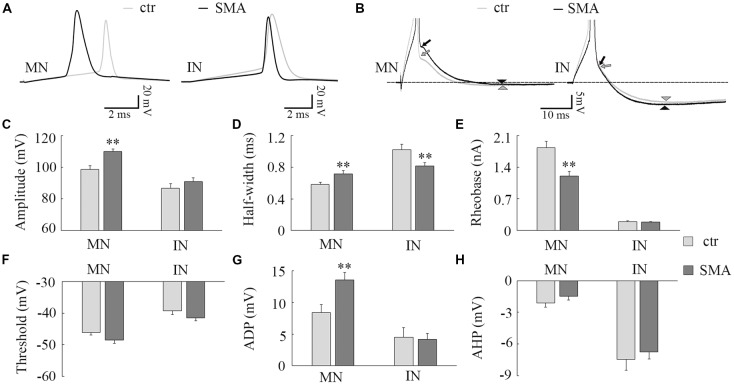
Differences in action potential (AP) properties in MNs and INs from SMA mice. **(A)** Representative recordings of APs from MNs (left) and INs (right) from control (gray) and SMA (black) mice. **(B)** Truncated AP recording from the same neuron in **(A)**, showing the site of calculation of afterdepolarization (ADP; arrow) and afterhyperpolarization (AHP; arrowhead) amplitudes. **(C–H)** For MNs, *n* = 13 cells for control, *n* = 17 cells for SMA from seven SMA mice and seven littermate controls, while for INs, *n* = 17 cells for control, *n* = 13 cells for SMA from six SMA mice and 11 littermate control mice. **(C)** Peak amplitude of APs. **(D)** Half-with of APs. **(E)** Rheobase current from SMA mice and controls. **(F)** The AP threshold trends toward hyperpolarized in both MNs (*P* = 0.06) and INs (*P* = 0.06) of SMA mice compared to controls. **(G)** ADP and **(H)** AHP in MNs and INs from SMA and control mice. **P < 0.01.

**Figure 3:**
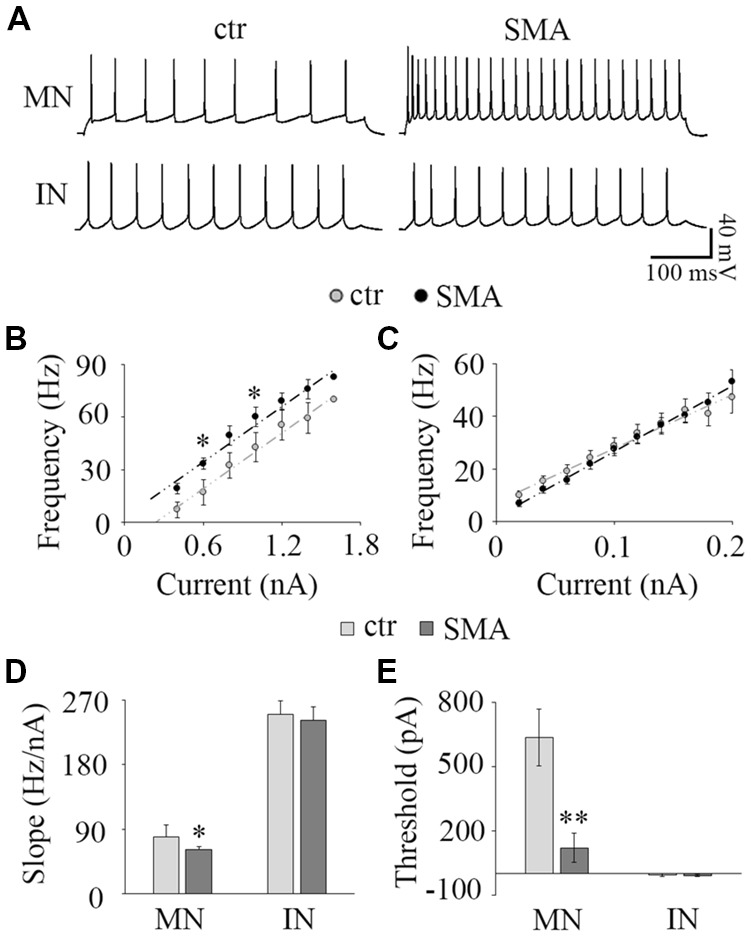
Input-output relationship. **(A)** The representative repetitive firing by 1.0 nA current injection in MNs (top) and 40 pA in IN (bottom) in control (left) and SMA (right) mice. **(B)** Average F-I of MNs; *n* = 15 for control and *n* = 15 for SMA. The firing frequency was significant higher at 0.6 nA and 1.0 nA current injections in SMA mice. **(C)** Average F-I for INs; *n* = 25 for control, *n* = 18 for SMA. **(D)** The slope of F-I representing the gain of the neurons in MNs and INs. **(E)** The threshold current of firing in MNs and INs. *P < 0.05, **P < 0.01.

**Figure 4:**
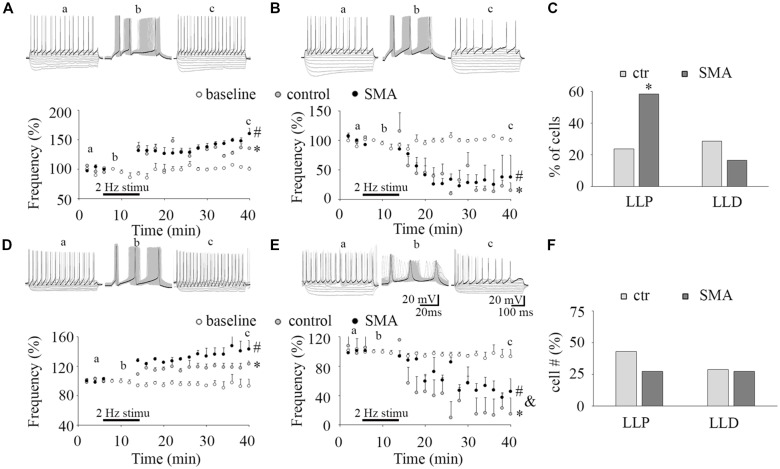
Long-lasting plasticity in intrinsic excitability is altered in MNs from SMA mice. **(A,D)** Long-lasting potentiation of intrinsic excitability (LLP-IE) of MNs **(A)** and INs **(D)**, top, representative traces of before conditioning stimulation (a), 2 Hz conditioning stimulation (b) initially evoked 3 APs (black trace in b), and recording after conditioning (c), black traces in “a” and “c” were the last steps for analysis. Bottom: average firing frequency with time, open circles are a baseline without the conditioning stimulation (*n* = 4, two from control, two from SMA mice for MNs; *n* = 4, three from control, one from SMA mice for INs), gray circles are control neurons with the conditioning stimulation, and black circles are SMA neurons with the conditioning stimulation. One-way ANOVA with Bonfferoni *post hoc* test, * control group was significantly potentiated compared with baseline without conditioning, *P* < 0.05 at all time points except at 20 and 26 min in MNs, and except at 14 and 36 min in INs. # SMA group was compared with baseline, *P* < 0.05 at all time points except at 22 min in MNs, and at all time points in INs. **(B,E)** Long-lasting depression of intrinsic excitability (LLD-IE) of MNs **(B)** and INs **(E)** top, representative traces of before conditioning stimulation (a), 2 Hz conditioning stimulation (b) evoked 3 APs initially (the black trace in b), and recording after conditioning (c), black traces in “a” and “c” were the last steps for analysis. Bottom: summary of the firing frequency with time, without conditioning stimulation (open circles) is the baseline, control with conditioning stimulation in gray circle and SMA with conditioning stimulation is the black circles. One-way ANOVA with Bonfferoni *post hoc* test, * control group was significantly depressed compared with baseline without conditioning, *P* < 0.05 at all time points except at 14 min in MNs, *P* < 0.01 at all time points in INs. # SMA group was compared with baseline, *P* < 0.05 at all time points except at 14 and 16 min in MNs, and at time points except at 14, 16, 18, 22 and 26 min in INs; & SMA neurons were less depressed compared with control at all time points except at 40 min. **(C)** Percent of MNs expressing LLP-IE and LLD-IE in controls (*n* = 21 cells) and SMA mice (*n* = 12 cells) from a total of five control mice and five SMA mice. **(F)** Percent of INs expressing LLP-IE and LLD-IE in controls (*n* = 14 cells) and SMA mice (*n* = 11 cells) from a total of seven control mice and seven SMA mice. *One-tailed distribution test, *P* < 0.05.

**Figure 5:**
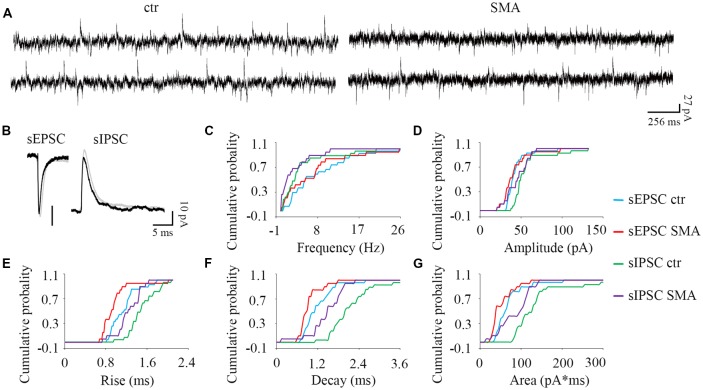
MNs from SMA mice compared to controls show more differences in inhibitory post-synaptic currents than excitatory post-synaptic currents. **(A)** Example traces of MNs from control and SMA mice showing both excitatory and inhibitory postsynaptic currents (EPSCs and IPSCs). **(B)** Average traces of sEPSCs and sIPSCs from one representative recording for MNs from control (gray) and SMA (black) mice. **(C–G)** Cumulative probability plots of results from recordings from 19 control and 15 SMA MNs was compared with K-S test (from a total of 10 control and 7 SMA mice). **(C)** Frequency of sEPSCs and sIPSCs did not change. **(D)** Amplitude of sIPSCs trended toward a decrease (*P* = 0.07), but sEPSCs were no different. **(E)** Rise time was significantly faster in both sEPSCs (*P* < 0.05) and sIPSCs (*P* < 0.05). **(F)** Decay time was significantly faster in both sEPSCs (*P* < 0.05) and sIPSCs (*P* < 0.01). **(G)** The charge transfer (area) was significantly lower in both sEPSCs (*P* < 0.01) and sIPSCs (*P* < 0.05).

**Figure 6:**
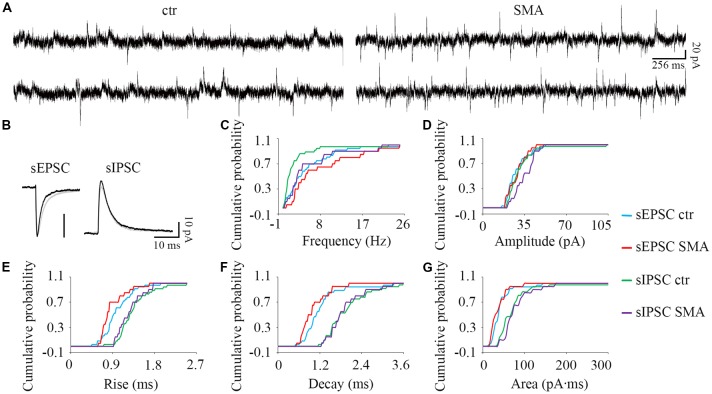
For INs there are few differences in post-synaptic currents from SMA mice compared to controls. **(A)** Example traces of INs from control and SMA mice showing both EPSCs and IPSCs. **(B)** Average traces of sEPSCs and sIPSCs from one representative recording for INs from control (gray) and SMA (black) mice. **(C–G)** Cumulative probability plots of results from recordings from 36 control and 20 SMA INs was compared with K-S test (from a total of 12 control and 7 SMA mice). **(C)** Frequency was significantly higher in sIPSCs (*P* < 0.01), but not in sEPSCs. **(D)** Amplitude of sEPSCs and sIPSCs. **(E)** Rise time was significantly faster in sEPSCs (*P* < 0.05), with no change for sIPSCs. **(F)** Decay time and **(G)** The charge transfer (area) of sEPSCs and sIPSCs did not change.

**Figure 7:**
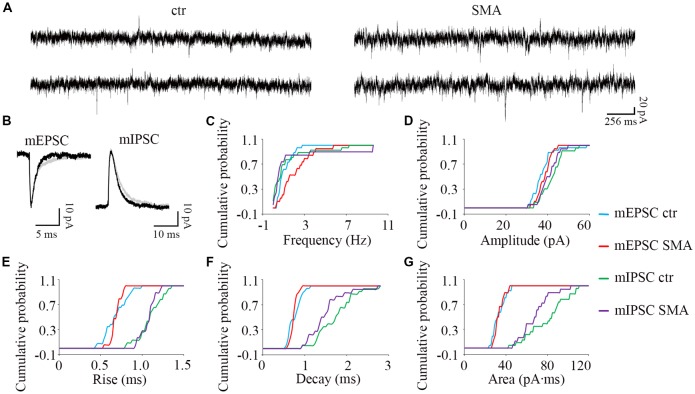
Higher frequency of miniature excitatory post-synaptic currents (mEPSCs) in MNs from SMNΔ7 mice. **(A)** Sample traces of miniature post-synaptic currents in MNs from control and SMA mice. **(B)** Average traces of mEPSCs and mIPSCs from one representative recording from a MN from control (gray) and SMA (black) mice. **(C–G)** Cumulative probability plots of results from recordings from 26 control and 19 SMA MNs was compared with K-S test (from a total of 4 control and 4 SMA mice). **(C)** Frequency of mEPSCs was significantly higher (*P* < 0.05), but no difference for mIPSCs. **(D)** The amplitude and **(E)** rise time of mEPSCs and mIPSCs were not different. **(F)** The decay time of mIPSCs (*P* = 0.07) has a trend to be faster, with no change for mEPSCs. **(G)** The average charge transfer (area) of mIPSCs (*P* < 0.05) was lower, with no change for mEPSCs.

**Figure 8:**
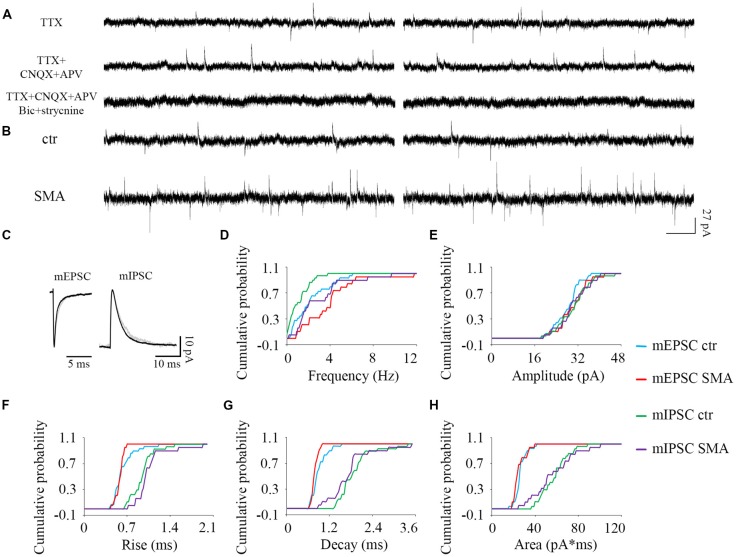
Higher frequency of both excitatory and inhibitory miniature post-synaptic currents in INs from SMNΔ7 mice.** (A)** The outward and inward miniature synaptic currents (Tetrodotoxin, TTX 1 μM) were validated with glutamatergic (CNQX 10 μM, AP-5 50 μM), GABAergic (bicuculline 10 μM) and glycinergic (strychnine 10 μM) receptor antagonists. Two traces are shown for each condition. **(B)** Sample traces of miniature post-synaptic currents in INs from control and SMA mice. **(C)** Average traces of mEPSCs and mIPSCs from one representative recording from an IN from control (gray) and SMA (black) mice. Two traces are shown for each condition. **(D–H)** Cumulative probability plots of results from recordings from 29 control and 19 SMA INs was compared with K-S test (from a total of 4 control and 4 SMA mice). **(D)** Frequency was higher in both mEPSCs (*P* < 0.05) and mIPSCs (*P* < 0.01). **(E)** Amplitude and **(F)** rise time of mEPSCs and mIPSCs did not change. **(G)** Decay time of mEPSCs was faster (*P* < 0.05), with no change in mIPSCs. **(H)** The average charge transfer (area) of mEPSCs and mIPSCs did not change.

### Analysis

Current-clamp recordings were analyzed with Clampfit 10.4 (Molecular Devices; Sunnyvale, CA, USA). Membrane input resistance (Rin) was calculated from averaged voltage deflections during the last 50.75 ms of the 500 ms hyperpolarizing current pulse injection. The membrane time constant (Tau) was derived from an exponential decay fit applied to the beginning 173.29 ms of the 500 ms current-evoked hyperpolarization steps when onset of the hyperpolarization did not evoke channel activation (Figures 1C,D). The capacitance of the neuron (C) was calculated by the measurement of Rin and tau (Tau = C*Rin). AP amplitude was measured from the first AP evoked. AP threshold was defined as the membrane potential at which *dV*/*dt* first exceeds 10 V/s (Fricker et al., 1999). The minimum current to evoke an AP was defined as the rheobase current. The baseline for determining the afterdepolarization (ADP) and the afterhyperpolarization (AHP) was the membrane potential before AP activation (−55 mV for INs and −65 for MNs). F-I relationships were fit linearly to get the current threshold, and the slope was defined as neuronal gain. The degree of long-lasting potentiation (LLP) and depression (LLD) was calculated by the average percentage of firing frequency from 20 min to 40 min compared to the average frequency before conditioning stimulation. The one-tailed *P* value for the proportion of percentage comparison was calculated in Excel. Voltage-clamp synaptic currents were analyzed with MiniAnalysis6.0.7 (Synaptosoft, Decatur, GA, USA). The data distribution was tested by the ratio of Skewness and Kurtosis to their standard error in Excel. The normality was rejected if the ratio is less than −2 or greater than +2. Significance in synaptic currents was tested by Kolmogorov-Smirnov (K-S) test, other data was tested with student *t*-test unless otherwise noted. Statistical significance was set at *P* < 0.05. The data shown represent means ± SEM.

## Results

### Resting Membrane Properties Suggest Increased Excitability in Both MNs and INs in SMNΔ7 Mice

A hyperexcitability of MNs has been reported in SMA mice (Mentis et al., 2011), in MNs derived from stem cell models of SMA (Simon et al., 2016), and iPSCs from severe SMA patients (Liu et al., 2015). Whether there are changes in the excitability of INs is unknown. In this study, we examined the excitability of both MNs and INs in the ventral horn of the lumbar spinal cords from post-natal day (P)6 to P10 SMNΔ7 mice (Figure 1). At rest, only 1 out of 31 MNs from control and no MNs (19) from SMNΔ7 mice showed spontaneous AP activity, while the majority of INs were spontaneously active (65% of control neurons and 71% of SMA neurons; Figures 1A,B,G). The frequency of spontaneous firing was higher in INs from SMNΔ7 mice compared to controls (9.7 ± 1.5 Hz vs. 6.5 ± 1.2 Hz, *P* < 0.05; Figures 1B,H). For MNs of SMNΔ7 mice, the resting membrane potential was hyperpolarized compared to control (−68.8 ± 1.0 mV vs. −64.3 ± 0.6 mV, *P* < 0.01; Figures 1A,I), while for INs, the resting potential was depolarized compared to MNs but was not significantly different in control compared to SMA (−52.1 ± 1.0 mV vs. −54.2 ± 1.0 mV; Figure 1I). The input resistance was significant higher in SMNΔ7 MNs compared to controls (44.2 ± 4.6 MΩ vs. 21.9 ± 2.0 MΩ, *P* < 0.01; Figures 1C,E,K) consistent with increased excitability, however the capacitance of SMNΔ7 MNs was lower compared to controls (264 ± 35 μF vs. 629 ± 84 μF, *P* < 0.01; Figure 1L). Current-voltage (I-V) curves used to calculate input resistance show the difference in intrinsic electrical properties in MNs from SMA mice and controls (Figure 1E), while INs show no difference (Figure 1F). The differences in input resistance and cell capacitance may have balanced out, as the membrane time constant was no different in MNs from SMNΔ7 mice compared to controls (10.5 ± 1.2 ms vs. 12.0 ± 0.1 ms; Figures 1C,J). For INs there was no difference between control and SMA neurons in input resistance (305 ± 33 MΩ vs. 304 ± 38 MΩ; Figures 1D,F,K), capacitance (control, 131 ± 27 μF; SMA, 127 ± 17 μF; Figure 1L) or time constant (control, 32 ± 4.6 ms; SMA, 34 ± 3.4 ms; Figures 1D,J). The input resistance of the INs in our recordings is somewhat lower than has been reported in other studies of the intrinsic properties of spinal INs (Zhong et al., 2006; Zhang et al., 2008). Possible reasons for this difference include the age of the animals (these recordings were made at P6–P10, while the earlier reports were of recordings made at P0–P3), the fact that in our studies fast synaptic transmission was not blocked as it was in the earlier studies, and differences in the populations of INs studied. Overall, our results indicate that although it is manifested in different ways, by increased spontaneous firing in INs, and by changes in the membrane properties in MNs, the membrane excitability of both MNs and INs appears to be increased in SMA mice.

#### Action Potential Properties Are Different in SMA Motor Neurons Compared to Controls, While Interneurons Show Fewer Differences Between Control and SMA

Consistent with other studies (Mentis et al., 2011; Liu et al., 2015; Simon et al., 2016), we found many differences in the AP properties of MNs in SMNΔ7 mice compared to their littermate controls. These differences included a larger AP amplitude (110 ± 1.6 mV in SMA vs. 99 ± 2.3 mV in control, *P* < 0.01; Figures 2A,C), a lower rheobase current (1.2 ± 0.1 nA in SMA vs. 1.8 ± 0.1 nA in control, *P* < 0.01; Figure 2E), and a trend toward a more hyperpolarized voltage threshold for APs in SMA mice compared to controls (−48 ± 1.1 mV vs. −46 ± 0.7 mV; *P* = 0.06; Figure 2F). Further, we also found a larger AP half-width (0.72 ± 0.04 ms vs. 0.58 ± 0.02, *P* < 0.01; Figures 2A,D), and greater amplitude in the ADP potential in SMA MNs compared to control (13.6 ± 1.2 mV vs. 8.4 ± 1.2 mV, *P* < 0.01; Figures 2B,G). The amplitude of the AHP is very small in MNs, and there was no difference between SMA and control MNs (−1.5 ± 0.4 mV vs. −2.1 ± 0.4 mV; Figures 2B,H).

There were some differences in the AP properties of INs from SMA mice. AP amplitude was not different between control and SMA mice (87 ± 2.8 mV vs. 91 ± 2.3 mV; Figures 2A,C), neither was rheobase current (0.19 ± 0.02 nA vs. 0.19 ± 0.01 nA; Figure 2E), ADP (4.5 ± 1.5 mV vs. 4.2 ± 0.9 mV, Figures 2B,G), nor AHP (−7.5 ± 1.0 mV vs. −6.7 ± 0.7; Figures 2B,H). However, the AP half-width was significantly smaller in SMA INs compared to control (0.82 ± 0.04 ms vs. 1.02 ± 0.07 ms, *P* < 0.01; Figures 2A,D). In addition, the AP threshold voltage was hyperpolarized in SMA INs compared to control neurons (−41.5 ± 0.8 mV vs. −39.2 ± 1.2 mV) although this trend did not reach significance (*P* = 0.06; Figure 2F).

These changes in AP properties indicate that there may be a difference in ion channel expression and/or function in SMA neurons (Liu et al., 2015). Overall, the differences in both passive membrane properties and the AP suggest an increased excitability of both MNs and INs in SMA model mice.

#### Changes in Input-Output Relationships in MNs and INs From SMNΔ7 Mice Compared to Controls

To assess how the changes in neuronal excitability in MNs and INs are expressed over a range of input currents, we studied the input-output relationships of MNs and INs in both control and SMA mice. The input-output (I-O) curve (also known as neuronal gain) was measured by the slope of a linear fit of the frequency-current (F-I) plot. In MNs, the frequency-current curve (F-I curve) was higher in SMA MNs compared to controls reflecting their increased excitability (Figure 3B). The threshold current was significantly lower in SMA mice compared to littermate controls (121 ± 70 pA vs. 635 ± 86 pA, *P* < 0.01; Figure 3E). However, the slope of the F-I relationship was lower in SMA (61.7 ± 3.5 Hz/nA in SMA vs. 79.6 ± 6.7 Hz/nA in control, *P* < 0.05; Figure 3D). The lower slope reflects the significantly higher firing frequency of SMA neurons compared to control at lower current injections (33.4 ± 3.0 Hz in SMA vs. 17.1 ± 4.6 Hz in control at 0.6 nA current injection, *P* < 0.05; 60.0 ± 5.5 Hz in SMA vs. 42.7 ± 7.2 Hz in control at 1.0 nA current injection, *P* < 0.05), but the differences were not significant at high current injections (Figures 3A,B). This is different from a previous study in P2 mice, that despite the increased input resistance, the firing rate was decreased in “SMA-affected MNs” (Fletcher et al., 2017). The reason for difference could be the age of the animals or/and the stage of SMA disease.

For INs there was no difference between the SMA mice and littermate controls in either F-I curve slope (242 ± 18 Hz/nA vs. 254 ± 21 Hz/nA; Figures 3A,C,D) or threshold current (−9.45 ± 5.2 nA vs. −6.37 ± 5.9 pA; Figure 3E). Since more than 65% of INs were spontaneously firing, the linearly fitted current threshold was slightly less than zero.

These results suggest that even though the excitability of MNs is greater in SMA mice, the input-output relationship is not as steep, so in SMA MNs firing frequency does not increase as much with increasing current as in control neurons. In contrast, the input-output relationship of INs was not different between control and SMA mice.

### SMA Increases the Long-Lasting Potentiation in the Intrinsic Excitability of MNs

The plasticity in intrinsic properties has been identified as a cellular correlate of learning in various brain areas including the hippocampus, neocortex, and cerebellum (Daoudal and Debanne, 2003). A long-lasting plasticity of intrinsic excitability has been reported in neurons in layer 5 of mouse barrel cortex neuron and plays a role in controlling sensory input efficiency (Mahon and Charpier, 2012). The mechanisms of the plasticity in intrinsic properties include changes in ion channel function (Beaumont and Zucker, 2000; Reyes, 2001), NMDAR (Daoudal et al., 2002), mGluR1 and mGluR5 (Sourdet et al., 2003; Ireland and Abraham, 2015), kainate receptors GluR6 (Melyan et al., 2002), CaMKII (Tsubokawa et al., 2000) and PKC (Ganguly et al., 2000). Long-lasting plasticity in intrinsic properties appears to share common features with the extensively studied synaptic plasticity. It is not known whether ventral horn neurons express a similar plasticity in intrinsic excitability and how it may be altered in SMA mice. To investigate this, we applied a 50 ms suprathreshold stimulation at 2 Hz frequency for 6 min to mimic sustained central pattern generator (CPG) activity in neonatal mouse spinal cord (Bonnot et al., 2002). This conditioning stimulus evoked three APs in each cycle (see “b” in Figures 4A,B,D,E). To measure the firing activity of the neuron while not distorting the neuronal activity by current injection, a test protocol of 10 equal current steps, five negative and five positive was applied. Before the 2 Hz conditioning stimulus (“a” in Figures 4A,B,D,E), the last step of positive current generated ~15 APs. After the conditioning stimulus (“c” in Figures 4A,B,D,E) the last positive current step generated increased numbers of APs in some neurons (LLP; Figures 4A,D) while in others the last current step generated fewer APs (long-lasting depression, LLD; Figures 4B,E). In control mice 24% of MNs expressed LLP (5 of 21 cells), and 29% expressed LLD, while in SMA mice 58% of MNs expressed LLP (7 of 12 cells), and 17% expressed LLD (Figure 4C). There was no difference between the SMA mice and littermate controls in the degree of LLP (129.2 ± 0.6% for control vs. 134.5 ± 4.5% for SMA) and LLD (25.74 ± 13.2% for control vs. 30.74 ± 8.7% for SMA). For INs, 43% of control neurons expressed LLP (6 of 14 cells), and 29% of neuron expressed LLD; while 27% of SMA neurons expressed LLP, and 27% of neuron expressed LLD (Figure 4F). There was no difference between the SMA mice and littermate controls in the degree of LLP (122 ± 5.8% for control vs. 135 ± 7.4% for SMA); but the degree of LLD was reduced in SMA mice (9.84 ± 2.5% for control vs. 55.9 ± 6.3% for SMA, *P* < 0.01). In conclusion, MNs from SMA mice were much more likely to express LLP than controls (one-tailed distribution test, *P* < 0.05) after the 2 Hz 6 min stimulation. No significant differences were observed between SMA and control INs in the percentage of cells with either LLP or LLD.

### Deficiencies in Presynaptic Inputs May Contribute to Excitability Changes and Trigger Compensatory Adjustments in Miniature Synaptic Current in Motor Neurons in SMA

#### Inhibitory Synaptic Input Is Reduced in MNs, but Increased in INs of SMNΔ7 Mice

While loss of excitatory synaptic input to MNs from proprioceptive afferents has been shown to be characteristic of SMN-deficient MNs (Mentis et al., 2011; Gogliotti et al., 2012; Martinez et al., 2012; McGovern et al., 2015), little is known about changes in synaptic transmission on other types of neurons. To investigate whether there are additional synaptic input impairments in MNs, and possibly synaptic changes in INs in SMNΔ7 mice, we used an approach to study both excitatory and inhibitory synaptic transmission simultaneously. By voltage clamping neurons at −55 mV, which is close to the resting membrane potential, it is possible to record both excitatory and inhibitory synaptic currents at the same time from the same neurons. This approach avoids the complex effects of receptor antagonists when receptor blockers are applied to isolate specific synaptic currents (Otis and Trussell, 1996; Brickley et al., 2001), and allows comparison of inhibitory and excitatory synaptic potentials in the same neurons.

Surprisingly given the known loss of proprioceptive excitatory inputs to MNs, for spontaneous excitatory post-synaptic potentials (sEPSCs) there was no difference between control and SMA MNs in frequency (7.88 ± 1.2 Hz vs. 6.71 ± 1.5 Hz) or amplitude (43 ± 2.5 pA vs. 41 ± 3.9 pA). For spontaneous inhibitory post-synaptic potentials (sIPSCs) the frequency was not different in SMA MNs compared to controls (2.71 ± 0.75 Hz vs. 4.37 ± 0.99 Hz), however the amplitude of sIPSCs trended smaller in SMA MNs compared to controls (44.9 ± 0.75 pA vs. 56.2 ± 4.2, *P* = 0.07; Figures 5A–D).

There were significant differences in the kinetics of the post-synaptic potentials. Rise time was significantly faster in SMA MNs for both sEPSCs (0.96 ± 0.07 ms vs. 1.15 ± 0.05 ms, *P* < 0.05) and sIPSCs (1.24 ± 0.06 Hz vs. 1.50 ± 0.05 ms, *P* < 0.05) compared to controls (Figure 5E). Similarly, the decay time was also significantly faster in SMA MNs for both sEPSCs (0.93 ± 0.07 ms vs. 1.19 ± 0.08 ms, *P* < 0.05) and sIPSCs (1.46 ± 0.11 ms vs. 2.21 ± 0.18 ms, *P* < 0.01) compared to controls (Figure 5F). Reflecting the faster rise and decay in MNs from SMA mice, the average charge transfer per post-synaptic current, as measured by the area of each synaptic current event, was lower in SMA MNs for both sEPSCs (52 ± 6.4 pA.ms vs. 69 ± 7.0 pA.ms, *P* < 0.01), and sIPSCs (85 ± 8.8 pA.ms vs. 150 ± 25 pA.ms, *P* < 0.05) compared to controls (Figure 5G).

For INs, there were fewer differences in spontaneous post-synaptic currents between SMA mice and controls. The frequency of sIPSCs was significantly higher in SMA INs (5.50 ± 1.3 Hz vs. 2.48 ± 0.66 Hz, *P* < 0.01; Figures 6A,C). There was no difference in the amplitude of sEPSCs in INs from SMA mice compared to controls (28.6 ± 1.7 pA vs. 28.4 ± 1.6 pA) or sIPSCs (control, 31.5 ± 2.4 pA; SMA, 35 ± 1.9 pA; Figures 6A,B,D). There was a faster rise time (0.89 ± 0.06 ms vs. 1.00 ± 0.05 ms, *P* < 0.05), but no change in the decay time (1.01 ± 0.09 ms vs. 1.22 ± 0.10 ms) in sEPSCs of SMA INs (Figures 6B,E,F). For sIPSCs, no differences were seen in INs from SMA mice vs. control mice for rise time (1.27 ± 0.06 ms vs. 1.34 ± 0.06 ms); decay time (1.97 ± 0.10 ms for control vs. 1.92 ± 0.12 ms for SMA). The charge transfer, as measured by the area of the synaptic currents, also was not different in INs from SMA mice for both sEPSCs (36.3 ± 4.5 pA.ms for SMA vs. 41.9 ± 4.5 pA.ms for control) and sIPSCs (77.6 ± 7.6 pA.ms for SMA vs. 78.7 ± 12 pA.ms for control; Figure 6G).

#### The Miniature Synaptic Transmission

To explore whether the changes we observe in synaptic transmission are due to changes in the presynaptic terminals or on the postsynaptic membrane, we recorded miniature synaptic currents in both MNs and INs with APs blocked by TTX (1 μM). Surprisingly, we found the frequency of mEPSCs was significantly increased in MNs from SMA mice compared to controls (2.07 ± 0.33 Hz vs. 1.06 ± 0.13 Hz, *P* < 0.05), although there was no difference in the frequency of mIPSCs (1.57 ± 0.67 Hz for SMA vs. 1.23 ± 0.37 Hz for control; Figures 7A,C). There were no significant differences in the amplitude or kinetics of mEPSCs between MNs from control and SMA mice (Figure 7; Table 1), however, for mIPSCs the decay time trended faster (*P* = 0.07; Figure 7F; Table 1) and the charge transfer for mIPSCs was significantly lower in SMA MNs (*P* < 0.05; Figure 7G; Table 1).

**Table 1:** Amplitude and kinetics of miniature post-synaptic currents in motor neurons (MNs) and interneurons (INs).

Neuron type	Current type	Condition	Amplitude (pA)	Rise time (ms)	Decay time (ms)	Charge transfer (pA.ms)
Motor neurons	mEPSCs	Control	37 ± 1.1	0.68 ± 0.03	0.76 ± 0.03	33 ± 1.3
		SMA	38 ± 0.8	0.68 ± 0.01	0.74 ± 0.02	40 ± 7.1
	mIPSCs	Control	42 ± 1.2	1.09 ± 0.03	1.73 ± 0.09	80 ± 4.1
		SMA	40 ± 1.1	1.07 ± 0.02	1.50 ± 0.10	68 ± 3.5*
Inter-neurons	mEPSCs	Control	28 ± 0.9	0.63 ± 0.03	0.89 ± 0.04	26 ± 1.0
		SMA	30 ± 1.3	0.58 ± 0.02	0.77 ± 0.02*	25 ± 1.4
	mIPSCs	Control	30 ± 1.2	0.94 ± 0.04	1.89 ± 0.09	58 ± 2.5
		SMA	30 ± 1.3	1.06 ± 0.06	1.74 ± 0.14	57 ± 4.6

Results from recordings from 26 control MNs and 19 SMA MNs, and 29 control INs and 19 SMA INs from a total of four control mice and four SMA mice. *P < 0.05.

The higher frequency of miniature EPSCs in SMΔ7 MNs, and the minimal differences in their kinetics is in contrast to what was observed with sEPSCs where the frequency was essentially the same in SMNΔ7 MNs compared to controls, while the rise and decay time were faster. On the inhibitory side, sIPSCs had a lower amplitude, faster rise and decay times and lower charge transfer in SMA MNs compared to controls, but the differences in amplitude and rise time were not observed for miniature IPSCs. These results suggest that for miniature EPSCs there may be a homeostatic adjustment in MNs of SMA mice, as has been reported for AP-independent synaptic currents in other neurons (Kavalali, 2015). This could not be the homeostatic effect of TTX blockage, since the fastest synaptic scaling has been reported at 4 h after TTX application (Ibata et al., 2008).

To examine the relative contribution of network-driven inputs in control and SMA neurons, the ratio of miniature to spontaneous EPSCs and IPSCs (mEPSC/sEPSC and mIPSC/sIPSC) was calculated by the average frequency, since the spontaneous and miniature synaptic currents were recorded from different neurons. The ratio of the average frequencies of mEPSC/sEPSC and mIPSC/sIPSC was 13% and 28% for control MNs and 31% and 58% for SMA MNs. These results suggest that AP-dependent synaptic inputs, both excitatory and inhibitory are diminished in SMA MNs relative to control neurons even more than the results with spontaneous EPSCs and IPSCs suggest. The likely decrease in the effectiveness of inhibitory synaptic inputs to MNs in SMA may be related to the apparent hyperexcitability of MNs that is observed in SMNΔ7 mice. This increase in the ratio of mEPSC/sEPSC and mIPSC/sIPSC as stated above suggests that there is a compensatory effect occurring on both excitatory and inhibitory synapses on MNs.

For SMNΔ7 INs, significant differences were observed compared to controls in the frequency of both mEPSCs (2.36 ± 0.32 Hz for controls vs. 3.87 ± 0.58 Hz for SMA, *P* < 0.05) and mIPSCs (1.08 ± 0.18 Hz for control vs. 2.95 ± 0.55 Hz for SMA, *P* < 0.01; Figures 8B,D). However, there were no differences in the amplitudes of either mEPSCs or mIPSCs (Figures 8C,E; Table 1). The rise times were not different for mEPSCs nor mIPSCs in SMNΔ7 INs compared to controls (Figure 8F; Table 1).The decay time was faster in SMNΔ7 INs for mEPSCs compared to controls (*P* < 0.05), but no differences were observed in mIPSCs (Figure 8G; Table 1). In spite of the differences observed in the rise and decay times of the mIPSCs and mEPSCs, no differences were observed between INs of control and SMNΔ7 mice in charge transfer (Figure 8H; Table 1). The higher frequency of action-potential-independent synaptic release in INs from SMNΔ7 mice for both mEPSCs and mIPSCs suggests a greater number of presynaptic contacts or/and increased release probability, with little change in the miniature post-synaptic potentials themselves.

The ratio of the average frequency for mEPSC/sEPSC and mIPSC/sIPSC in INs was higher in SMA INs compared to controls (49% and 54% compared to 40% and 44%). The increase in frequency of sIPSCs in SMA INs suggests an increase in inhibitory synaptic inputs onto a subgroup of INs that may depress the activity of the preMNs, potentially contributing to MN hyperexcitability (Simon et al., 2016). Both AP-dependent and spontaneous release may contribute to this effect.

## Discussion

This study provides a detailed illustration of the membrane properties and synaptic currents of both MNs and ventral horn INs of SMNΔ7 mouse pups compared to littermate controls, and represents the first report of the electrophysiological properties of SMA-affected INs. Although we were not able to sort INs into different types based on their function, our results suggest that spinal motor neural networks are substantially altered in SMA mice compared to controls, and that the differences may contribute to the deficiencies in MN output that have been observed (Kong et al., 2009).

### The Mechanisms Under Neuronal Intrinsic Property Change

The selective loss of alpha MNs in SMA has been confirmed in patients (Crawford and Pardo, 1996) and mouse models (Ling et al., 2010; Mentis et al., 2011; McGovern et al., 2015; Powis and Gillingwater, 2016), and MNs in mouse models also show a characteristic hyperexcitability (Mentis et al., 2011). Recent work with a stem cell-based model of the motor circuit suggests that, while neuronal death is a cell-autonomous effect of low SMN expression in MNs, the hyperexcitability likely results from the MN response to defects in premotor INs arising from low levels of SMN expression in those neurons (Simon et al., 2016). However, no other study has investigated alterations in the function of INs with low SMN expression. In our recordings from spinal cord slices we were not able to distinguish INs by type, inhibitory or excitatory, and this undoubtedly concealed and diluted differences in the activity of different types of INs. Still, we observed an overall increase in the activity of INs. The majority of ventral horn INs from control and SMA mice showed spontaneous activity, while the frequency of spontaneous AP firing was significantly higher in INs from SMA mice compared to controls. The smaller half-width of APs in the SMA INs compared to controls may contribute to their increased firing frequency. New tools and approaches to classify the different functional types of INs, might identify a specific type of IN whose activity and properties are substantially altered by low SMN expression.

We observed an increase of MN excitability in slices from SMA mice compared to controls, with a hyperpolarized AP threshold, increased input resistance and reduced rheobase current for initiation of APs, and this is also consistent with previous studies in mice (Mentis et al., 2011), stem cell models (Simon et al., 2016), and iPSC cells from severe SMA patients (Liu et al., 2015). While previous studies have suggested that the somatodendritic area of SMA MNs is not different from wild type (Mentis et al., 2011; Simon et al., 2016), we observed a significant decrease in membrane capacitance in SMA mice, suggesting a decrease in the soma area. The differences between our results and earlier findings could be related to differences in the age of the mice at the time of recording. At postnatal day four (P4), the average soma area was shown to be no different in MNs from SMA mice compared to controls (Mentis et al., 2011), but has been shown to be lower in SMA mice at P7–P8 (Tarabal et al., 2014). Our recordings were conducted in P6–P10 mice, and since MN loss is progressive with age (Kong et al., 2009; Tarabal et al., 2014) and it is likely that bigger MNs are lost earlier, the average somatodendritic area of the surviving MNs would be expected to decrease with age.

Interestingly, in spite of the MN hyperexcitability we observed, we also found that the resting membrane potential in SMA MNs was hyperpolarized compared to controls, while other studies have reported no change in resting membrane potential (Mentis et al., 2011; Simon et al., 2016). This difference could be related to the age of the mice at recording, and differences between mouse neurons and the stem cell model. The hyperpolarized membrane potential we observe may contribute to the increased amplitude of APs in SMA MNs, since more voltage-gated sodium channels will be released from voltage-dependent inactivation and in the channel pool that can readily open with depolarization. Greater availability of sodium channels may also contribute to the higher amplitude of APs as reported in SMN-deficient induced pluripotent stem cells (Liu et al., 2015). The widening of the AP and larger ADP in SMA MNs suggests that multiple channels play a role in the hyperexcitability of MNs in SMA, as has been observed in other types of neurons (Bean, 2007). Application of the quantitative polymerase chain reaction (q-PCR) technique in the stem cell model may validate changes in the specific channel expression in the future (Maeda et al., 2014; Liu et al., 2015; Simon et al., 2016). Surprisingly, in spite of the higher excitability of MNs in SMA, there is a decrease in the input-output relationship, so that the difference in firing frequency between SMA MNs and controls narrows at higher levels of injected current. This may be related to the deficiency of output from MN in SMA.

### Differences in the Plasticity of Intrinsic Electrical Properties Hint at a Deficiency in the Motor Network in SMA Mice

Even though alterations in the plasticity in the intrinsic electrical properties of neurons can fundamentally alter the input-output properties of neuronal networks in CNS disorders, it receives limited attention (Beck and Yaari, 2008). Long-lasting plasticity in the intrinsic excitability of neurons has been reported in the somatosensory cortex (Mahon and Charpier, 2012), hippocampus (Xu et al., 2005) and deep dorsal horn neurons (Kim et al., 2008). Excitability changes in MNs have been reported after chronic activity *in vivo* (Beaumont and Gardiner, 2003; Cormery et al., 2005; MacDonell et al., 2012) as well as after prolonged activation in slices (Lombardo et al., 2018), however LLP of the intrinsic excitability has not been studied in detail in spinal MNs. In this study, we investigated the expression of this form of plasticity and how it is altered in ventral horn neurons from SMA mice. For 6 min we applied a 2 Hz suprathreshold stimulation to mimic the spinal CPG activation in neonatal mice (Bonnot et al., 2002), producing three APs for each 50 ms stimulation. After this stimulation, the percentage of neuron expressing LLP of the intrinsic excitability was more than doubled in SMA MNs compared to controls, suggesting that the input-output gain of the neurons in response to this CPG activity-like conditioning stimulation is higher in SMA mice. In contrast, without the conditioning stimulation, the input-output relationship measured by the slope of the F-I curve is lower in SMA MNs. These results suggest that chronically deficient inputs may play a role in the decline of the MN output that has been observed in SMA mouse models (Kong et al., 2009), and is also is consistent with the finding that MN firing varies with afferents and descending inputs in rats (MacDonell et al., 2012). Interestingly, in INs, we observed a trend in the opposite direction for LLP in SMA neurons compared to controls. The percentage of INs expressing LLP trended lower in SMA than in controls, indicating that there may be a reduced input-output relationship in the IN network of SMA mice. These results suggest that there are additional impairments of the motor network in SMA involving the spinal IN network.

### Changes in Presynaptic Inputs May Be Related to Deficient MN Output in Mouse Models of SMA

MN death is the histopathologic hallmark of SMA, while loss of afferent inputs to MN has been detected much earlier, even at embryonic stages (Tarabal et al., 2014). As early as P4, MNs from SMA mice have been shown to have reduced responsiveness to proprioceptive input and decreased number and function of glutamatergic synapses (Mentis et al., 2011). A decline in GABAergic inputs to MNs has been reported to start much later (P7–P8) than the glutamatergic loss (Tarabal et al., 2014), while in a stem cell model, SMN deficiency in INs caused a loss of glutamatergic but not GABAergic synapses on MNs (Simon et al., 2016). Interestingly, in spite of the loss of excitatory synapses on SMA MNs detected with immunohistochemistry, our electrophysiological recordings showed no difference in either the frequency or amplitude of AP-driven excitatory synaptic currents in SMA MNs. Our study of synaptic currents in MNs from P6–P10 mice showed more differences primarily in inhibitory synaptic currents in MNs of SMA mice compared to controls. We report a smaller reduction of excitatory synaptic currents compared with other studies that focused on proprioceptive synapses (Mentis et al., 2011; Fletcher et al., 2017). MNs in the spinal cord receive inputs from the local spinal network, descending pathways in addition to the sensory neurons, and they receive extensive projections from the brain stem (Rekling et al., 2000). The sEPSCs we record are the summation of all excitatory inputs to MNs. The lack of difference in overall sEPSC frequency suggests that excitatory inputs from other pathways either are not changed or change in the opposite direction that masks the reduction in input from proprioceptive synapses in SMA mice. For inhibitory post-synaptic currents, there was no decrease in frequency, but a reduction in their average amplitude as well as changes in their kinetics, that would have the effect of reducing the inhibitory tone of inputs to MNs.

The faster rise time and decay time in both excitatory and inhibitory post-synaptic potentials may be related to a decrease in the number of release sites or to changes in the type of receptor carrying post-synaptic current. For example, a change in NMDA vs. AMPA glutamatergic receptors (Rekling et al., 2000) or GABA vs. glycine receptors (González-Forero and Alvarez, 2005), would result in different kinetics for the current reflecting the differences in the underlying channels. For INs, the only significant difference between SMA neurons and controls for action-potential driven synaptic currents was in the frequency of inhibitory post-synaptic potentials, which were increased in SMA neurons. Along with the increase in spontaneous firing of INs, this suggests that inhibitory IN network may be more active in SMA mice.

For the miniature synaptic currents which are AP-independent, surprisingly, the frequency of excitatory currents was higher in SMA MNs. This suggests an increase in presynaptic release of glutamate, which is opposite what would be expected from the decrease in synaptic contact numbers reported in other studies (Mentis et al., 2011; Tarabal et al., 2014; Simon et al., 2016). This suggests that there is a compensatory adjustment in AP-independent glutamate release onto MNs. A similar increase in the frequency of miniature synaptic events vs. spontaneous synaptic events has been reported after traumatic injury in mossy cell of dentate gyrus (Howard et al., 2007). In addition, more studies are suggesting that spontaneous release is very different from AP-driven release with different machinery, vesicle release pools, even different postsynaptic receptor subtypes (for review see Kavalali, 2015). The loss of synaptic inputs to SMA MN may activate the homeostatic function of spontaneous release to compensate for declines in synaptic function, perhaps increasing release probability at the remaining excitatory release sites. This may also be related to the apparent preservation of the frequency of excitatory post-synaptic potentials in MNs even as excitatory synaptic contacts are lost.

For INs, the frequency of miniature synaptic currents was higher in SMA mice for both excitatory and inhibitory post-synaptic currents. Unlike for MNs, it is not clear if loss of synaptic inputs onto INs is part of the pathophysiology of SMA, so the increase in the frequency of miniature post-synaptic currents may be related either to an increase in release sites on the INs or a higher release probability in SMA neurons compared to controls. A higher number of release sites could arise from disease-related changes in INs other than the preMNs that directly contact MNs. Our hypothesis is that one or more types of inhibitory activity are increased, which then limits both excitatory and inhibitory IN inputs to MNs after SMA. As mentioned earlier, new tools and approaches are necessary to dissect the neuron types that are altered in the SMA model.

### The Adjustment of the Plasticity in Intrinsic Properties

Plasticity in the intrinsic electrical properties of neurons has been observed in many CNS disorders (Beck and Yaari, 2008). Changes in AP threshold have been observed in animal models of chronic pain (Wang et al., 2007), and the modification of somatic ion channels such as sodium, A-type of potassium, and T-type of calcium channels contribute to the increase of the excitability of DRG neurons (Hu et al., 2006; Tan et al., 2006; Jagodic et al., 2007; Wang et al., 2007). Similar mechanisms may be operating in SMA MNs, as the expression of Kv2.1 potassium channels has been shown to be reduced in MNs of SMA mice (Fletcher et al., 2017), while a Na^+^ current was increased in an iPSC model for SMA MNs (Liu et al., 2015). Changes in sodium and potassium channels may also contribute to the differences in amplitude and half-width between the APs of SMA MNs and those of control animals.

Changes in neuronal firing mode have been reported in epilepsy (Sanabria et al., 2001), stress (Okuhara and Beck, 1998), chronic pain, (Cummins et al., 2007; Hains and Waxman, 2007) and experimental allergic encephalomyelitis (Saab et al., 2004). In addition, the large size of the AP ADP in SMA MNs has the potential to change the firing properties of the neurons. In other neurons, the interplay between axo-somatic persistent sodium channels and M-type potassium channels modulates the size of spike ADPs and the firing mode of the neurons (Beck and Yaari, 2008), and calcium channels that underlie the ADP contribute to the firing mode change of CA1 neurons in a chronic epilepsy animal model (Yaari et al., 2007). Our previous study of the contribution of M-type potassium channels to activity-dependent changes in the intrinsic properties of MNs (Lombardo et al., 2018) suggests that M-channels could be a target for plasticity in the intrinsic properties of MNs in SMA. The increased rate of LLP in SMA MNs, accompanied by the decrease in LLP in SMA INs suggests that changes in the plasticity of intrinsic properties may affect multiple types of neurons in SMA. These changes suggest that there is an adjustment in the intrinsic properties of neurons in the motor network that may either contribute to the pathophysiology of SMA or partially compensate for it.

## Conclusion

Synaptic plasticity coexists and functionally interacts with the plasticity of intrinsic properties in most neurological disorders (Beck and Yaari, 2008). From this study, it is clear that a compensatory adjustment happens in both intrinsic properties and synaptic inputs of the MNs in SMA mice. The plasticity of the neuro-motor system in the context of spinal cord injury (SCI) has been studied extensively (Raineteau and Schwab, 2001), and activity-based, pharmacological and gene-delivery approaches to facilitate that plasticity are under intense investigation as a way to enhance the recovery after SCI (Onifer et al., 2011). However, the potential of plasticity mechanisms to aid the survival of MNs and sustain the function of the motor network in SMA has attracted much less attention. More research is needed to determine if changes that we have observed in the intrinsic and synaptic properties of MNs and INs contribute to the pathophysiology or are part of a compensatory response that helps preserve the function of the motor network. If the plasticity observed in SMA MNs is beneficial, it may be a useful target for novel therapeutic approaches (Bora et al., 2018). The combination of the current approaches that target increasing SMN levels (Bowerman et al., 2017) with other approaches that prolong the survival of MNs and enhance their output, may be of tremendous benefit to SMA patients.

## Author Contributions

JS and MH contributed to design of the experiment. JS performed all experiment and statistical data analysis. JS and MH contributed to writing of the manuscript.

## Conflict of Interest Statement

The authors declare that the research was conducted in the absence of any commercial or financial relationships that could be construed as a potential conflict of interest.

## Abbreviations

ACSF: artificial cerebral spinal fluid
ADP: afterdepolarization
AHP: afterhyperpolarization
AMPA: α-amino-3-hydroxy-5-methyl-4-isoxazolepropionic acid
AP: action potential
CPG: central pattern generator
EPSC: excitatory postsynaptic current
F-I curve: frequency-current curve
GABA: γ-Aminobutyric Acid
IN: interneuron
IPSC: inhibitory postsynaptic current
LLD: long-lasting depression
LLP: long-lasting potentiation
MN: motor neuron
NMDA: N-Methyl-D-aspartic acid or N-Methyl-D-aspartate
SCI: spinal cord injury
SMA: spinal muscular atrophy
SMN: survival motor neuron
TTX: Tetrodotoxin
VAChT: Vesicular acetylcholine transporter
VGLUT: vesicular glutamate transporter.
